# Supplementary material for: One-dimensional electron gas in strained lateral heterostructures of single layer materials
Source: Sci Rep. 2017 Jun 28;7:4316. doi: 10.1038/s41598-017-03880-w (PMC5489521; doi:10.1038/s41598-017-03880-w)
Supplement: Supplementary file 1 — Supplementary information [file 41598_2017_3880_MOESM1_ESM.pdf]

One-dimensional electron gas in strained lateral heterostructures of single layer materials (supplementary information)

O. Rubel[1]

[1]*Department of Materials Science and Engineering, McMaster University, 1280 Main Street West, Hamilton, Ontario L8S 4L8, Canada*

Figure 1 illustrates the potential energy profile across a strained MoS_2/WS_2 lateral heterojunction. The results are shown for the external strain of $\epsilon_1 = 0.1$. The electric field magnitude $|E|$ varies in the range of $7.5 - 9.3$ meV/Å. The average field is approximately 8.2 meV/Å. Note that the electric field does not vanish in the vacuum region that separates periodic images of the structure, even though the thickness of the vacuum region is 24.6 Å.

Figure 2 shows the induced electric field and the band gap of a lateral heterostructure as a function of an external strain ϵ_1. The electric field increases proportionally to the strain, while the band gap decreases at the same time. Results of the continuum model agree with the DFT calculations up to the critical point $\epsilon_1 \approx 0.15$ where the band gap vanishes. Here the electric field reaches the maximum and saturates being screened by the metallic states of the emerged 1DEG. It is expected that the critical value of the strain or the induced electric field corresponding to the onset of the metallic states scales inversely with the length of the heterostructure L.

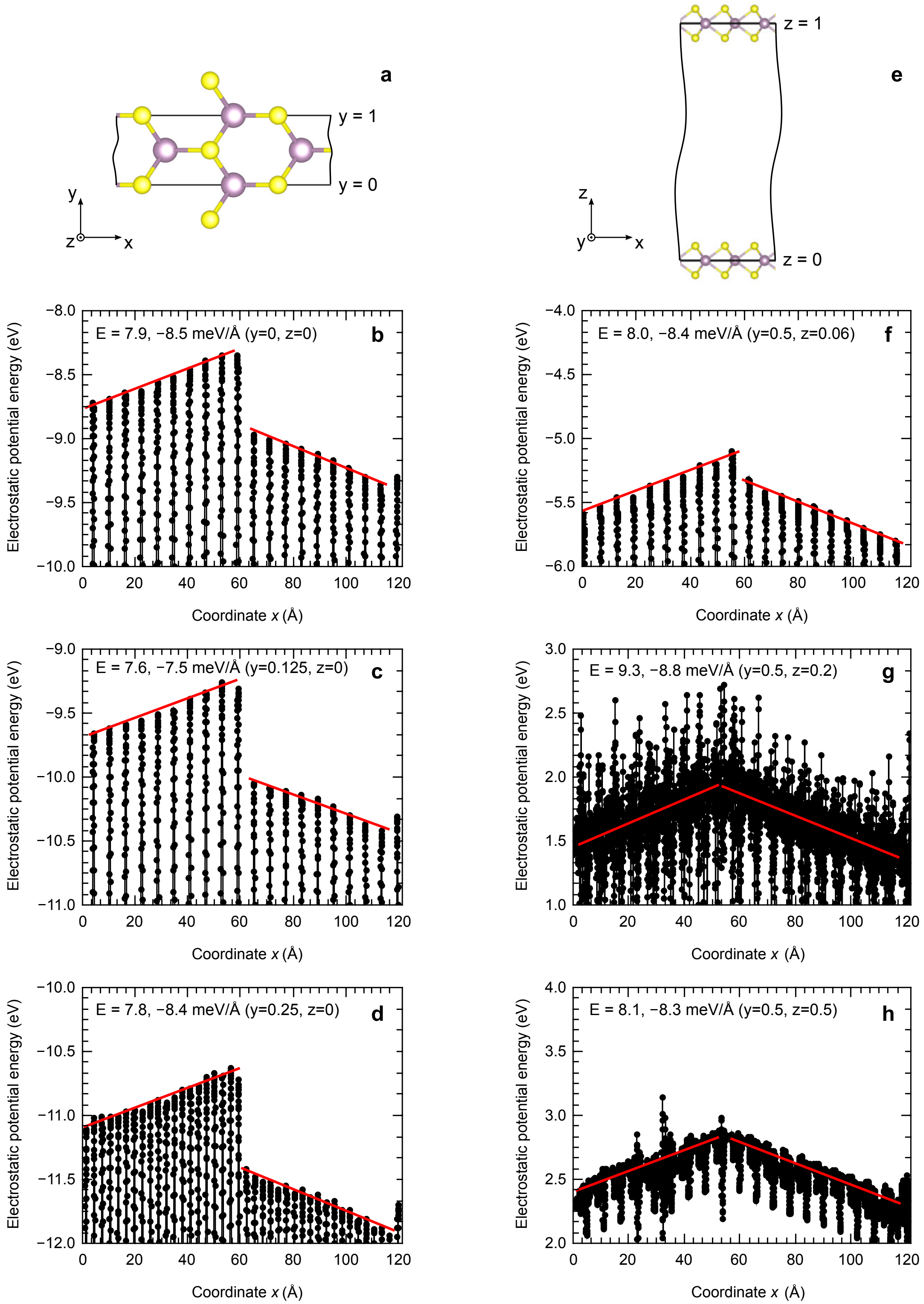

FIG. 1: Electrostatic potential energy profile across the MoS_2/WS_2 strained lateral heterojunction ($\epsilon_1 = 0.1$): (a–d) scans are taken along lines with varying y-coordinate within the plane of the MoS_2/WS_2 structure; (e–h) scans are taken along lines with varying offset z from the plane. The electric field E is evaluated as a slope of the potential energy profile.

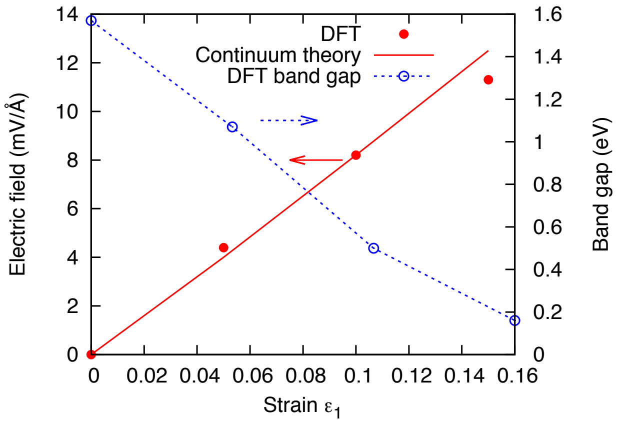

FIG. 2: Induced electric field and the band gap in the 11 nm long lateral MoS_2/WS_2 heterostructure as a function of an external strain ϵ_1.
